# How many species are there on Earth? Progress and problems

**DOI:** 10.1371/journal.pbio.3002388

**Published:** 2023-11-20

**Authors:** John J. Wiens

**Affiliations:** Department of Ecology and Evolutionary Biology, The University of Arizona, Tucson, Arizona, United States of America

## Abstract

How many species are there on Earth? Projections have ranged from the millions to the trillions. This Perspective looks at how a 2011 PLOS Biology study transformed this field and what is still to be done to find a definitive answer.

## Teaser

How many species exist on Earth? Projections range from millions to trillions. A 2011 paper in *PLOS Biology* provided a comprehensive estimate of 9 million.

This article is part of the *PLOS Biology* 20th Anniversary Collection.

In 2010, Robert May [1] pointed out an embarrassing truth about modern science. Even as we invest huge amounts of time, money, and effort to find life on other planets, we still do not know how much life (i.e., how many species) is on our own. Although “do not know” might sound like hyperbole, estimates have ranged wildly, from 2 million [2] to 3 trillion [3].

Countering this embarrassing situation, a study by Mora and colleagues [4] represented a transformative advance. This paper has since become the “default” estimate of the number of living species on the plant (8.75 million), even though approximately 80% of those species are hypothetical. Here, I discuss why this paper represented important progress and how we might continue to move forward in estimating this fundamental number in science.

What was so special about the study by Mora and colleagues? When it was published, various other biodiversity projections existed, based on various approaches. Two major advances were that it was statistically based and taxonomically comprehensive. For example, many previous estimates relied on expert opinion or questionable assumptions [4]. Furthermore, most earlier studies focused on a particular group (e.g., insects or plants), rather than all of life. Thus, most studies did not even attempt to estimate the number of species on Earth.

The approach of Mora and colleagues [4] involved two main steps. First, they used biodiversity databases to plot the numbers of new higher taxa (genera to phyla) described in each major group over time, in order to project the value at which these numbers might asymptote (i.e., stop increasing) in the future. Second, they regressed these estimated asymptote numbers of higher taxa at each taxonomic level (e.g., the number of phyla) against a ranking of taxonomic levels (1 to 6, from phylum to species) and then used this regression model to extrapolate the unknown number of species in each group. Using this approach, they estimated that there are about 7.8 million animal species, 298,000 plants, 611,000 fungi, and 63,900 protists. They estimated relatively few prokaryotes (10,000 bacteria and 500 archaea). Overall, they projected that there are 8.75 million living species, of which only 1.2 million were described (including approximately 0.95 million animal species). Mora and colleagues validated this approach by comparing their projections [4] to those from expert opinion [5]. They did this for 18 groups in which total species numbers (described and undescribed) were considered “relatively well known.” They found a strong relationship between these estimates.

The study by Mora and colleagues [4] has become very widely cited and used, and deservedly so. Yet, it would be problematic to treat their estimates as the final word on global biodiversity and especially to reject new estimates simply because they are larger. Mora and colleagues generally did not incorporate the vast species numbers revealed through molecular analyses (e.g., metabarcoding). Below, I review published diversity projections for several key groups that are dramatically larger than those from Mora and colleagues, with species revealed by molecular data largely driving these bigger estimates (Table 1).

**Table 1:** Contrasting estimates of species richness from Mora and colleagues with projections that incorporate molecular data.

Group	Mora and colleagues [4] estimate of species richness	Alternative estimate of species richness	Source of recent estimate
Bacteria	9,700	2–4 million to 3.2 trillion	[3,6]
Protists	63,900	1–10 million	[7]
Fungi	611,000	6.3 million	[8]
Insects	<5.6 million	21.1 million	[9]
Insect-associated taxa	<5.6 million	Approximately 50–90 million each of animals, protists, and fungi; many more bacteria (hundreds of millions)	[9,10]

Mora and colleagues [4] estimated approximately 10,000 bacterial species (roughly the number of described species). They acknowledged that these projections were likely underestimates. Yet, prokaryotes may be a major driver of Earth’s overall species richness. Recent studies have estimated a staggering range of species numbers for bacteria, from low millions [6], to hundreds of millions [10], to low trillions [3]. All were based on extrapolations from molecular studies. Clearly, controversies about global biodiversity cannot be resolved without better resolving bacterial richness.

For protists, Mora and colleagues [4] estimated 63,900 species. Yet, Adl and colleagues [7] estimated 1.2 to 10 million species, almost all in *Apicomplexa* (including the malaria-causing *Plasmodium*). This projection was based on the “number of unknown DNA sequences found in environmental samples.” However, no specific methodology was given for these extrapolations, which makes them difficult to rigorously evaluate.

For fungi, Mora and colleagues estimated 611,000 species. Nonetheless, estimates of global fungal diversity from environmental sequencing methods have ranged into the millions for years now [11]. A recent study [8] combined estimates from 335 million comparable nucleotide sequences from >200 studies. They found 1.1 million putative species, after excluding a remarkable 9.5 million potential species each represented by a single sequence. These putative species were mostly *Ascomycota* (57%; yeasts and relatives) and *Basidiomycota* (37%; mushrooms and relatives). They extrapolated from this sampling to conservatively project 6.3 million fungal species.

Many studies have estimated the total number of insect species. Their projections have been comfortingly similar for decades, often around 6 million species [12]. This number is similar to the 5.6 million terrestrial animal species estimated by Mora and colleagues [4]. However, these estimates have not explicitly incorporated morphologically cryptic species revealed by molecular analyses. Recent analyses suggest that each insect species initially delimited by morphology might conceal (on average) 3.1 cryptic species [9]. Combined with projections of approximately 6 million morphology-based insect species, this yields estimates of approximately 20 million insect species [9]. Nevertheless, these extrapolations could be overestimates for two main reasons. First, large-scale barcoding studies have not found as many cryptic species as have studies of individual species [12]. However, it is unclear if this discrepancy is caused by limited geographic sampling within species in large-scale barcoding studies or instead by biased selection of species for studies of individual species (but see [9]). The second problem in these extrapolations is the assumption that undescribed insect species harbor as many cryptic species as described species. Instead, undescribed species might be more narrowly distributed than typical described species and thus might contain fewer cryptic species. Both problems require further study.

Another complication is that the diversity of some taxonomic groups might depend on other organisms. A review suggested that each insect species might host (on average) a unique species of mite, nematode, apicomplexan protist, and microsporidian fungus, and several bacteria [10]. These inferences were based on case studies that focused on species in these groups hosted by closely related insects. When combined with larger projections of insect diversity, these insect-associated species could push global biodiversity past 100 million species, with tens of millions from these five groups. Some readers might reasonably be squeamish about projecting such enormous numbers based on relatively few case studies. What is therefore needed are additional studies of closely related insect species to document the number and specificity of their host-associated species from these five groups (and possibly others).

In summary, Mora and colleagues [4] made a transformative contribution to the study of Earth’s biodiversity. They combined existing biodiversity databases with rigorous statistical methods to produce one of the first comprehensive estimates of species numbers spanning all major groups. Yet, new molecular data are dramatically increasing richness estimates for many of these groups (Table 1). Our estimates of global biodiversity should continue to evolve as they incorporate these new types of data.

Unfortunately, estimates of global biodiversity may soon be changing fundamentally in another way. Global biodiversity is now facing numerous threats. The most important ones may be habitat destruction and overexploitation [13], and climate change can threaten even protected species in well-preserved habitats [14]. Soon, we may not be estimating how many species there are on Earth. We will be estimating how many there were.
